# Déformation de Madelung à propos d'un cas et revue de la littérature

**DOI:** 10.11604/pamj.2016.23.137.9002

**Published:** 2016-03-25

**Authors:** Kouassi Kouame Jean Eric, Yao Loukou Blaise, Krah Koffi Leopold, Sery Bada Justin Léopold Niaore, M'bra Kouamé Innocent, Assere Yao Aboh Ganyn Robert Arnaud, Kodo Michel

**Affiliations:** 1 Service d'Orthopédie, Traumatologie, CHU de Bouaké, Côte d'Ivoire

**Keywords:** Maladie de Madelung, Ulna, radius, Madelung's disease, ulna, radius

## Abstract

La maladie de Madelung est une déformation du poignet due à une atrophie de la partie médiale du cartilage de croissance distal du radius. Il en résulte une subluxation antéro-médiale du carpe,limitant les amplitudes articulaires. Cette dysplasie osseuse est rare et représente 1,7% des anomalies congénitales. Les auteurs rapportent un cas bilatéral chez une fille de 21 ans. L’étude de cette observation nous a permis d’étudier la fréquence, les signes et les moyens de son diagnostic ainsi que les mesures thérapeutiques adéquates afin de pouvoir répondre aux attentes des patientes.

## Abstract (en)

Madelung's disease is a wrist deformity due to atrophy of the medial portion of the distal radial growth plate. It results in carpal anteromedial subluxation, limiting the range of motion. This skeletal dysplasia is rare and represents 1.7% of birth defects. The authors report a bilateral case in a 21 year old girl. This observational study allowed us to study the frequency, the signs and the means of diagnosis as well as adequate therapeutic measurements, in order to meet the expectations of patients.

## Introduction

La déformation de Madelung a été décrite en 1878, comme une « subluxation palmaire, spontanée et progressive, du poignet » rarement symétrique, à prédominance féminine [1]. Elle est rare et représente 1,7% des anomalies congénitales [2]. Nous rapportons un casdécouvert à l’âge adulte.

## Patient et observation

**Figure 1:**
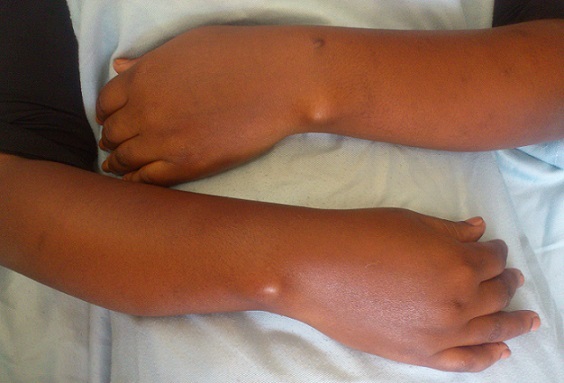
Saillie postérieure bilatérale de la tête ulnaire

**Figure 2:**
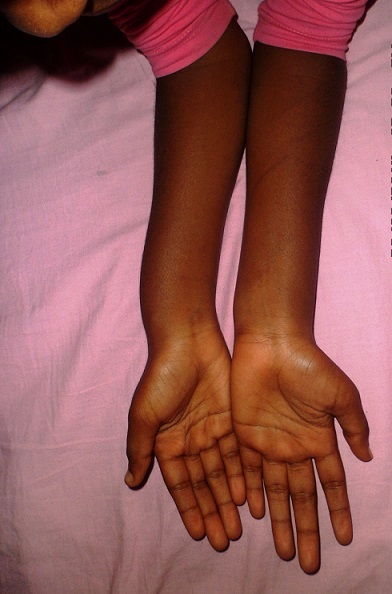
Courbure du radius et un déjettement palmaire du carpe à droite

**Figure 3:**
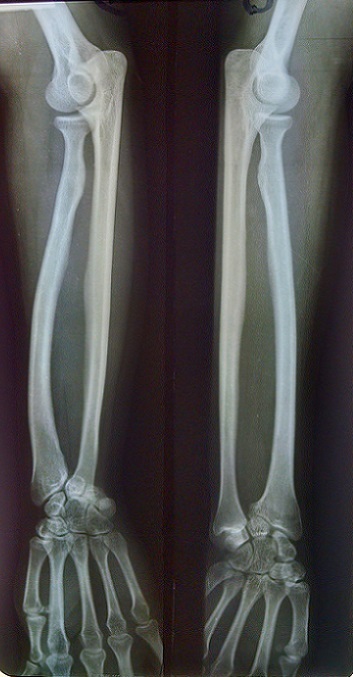
Radiographie comparative de l'avant-bras vue de face d'une déformation de Madelung

**Figure 4:**
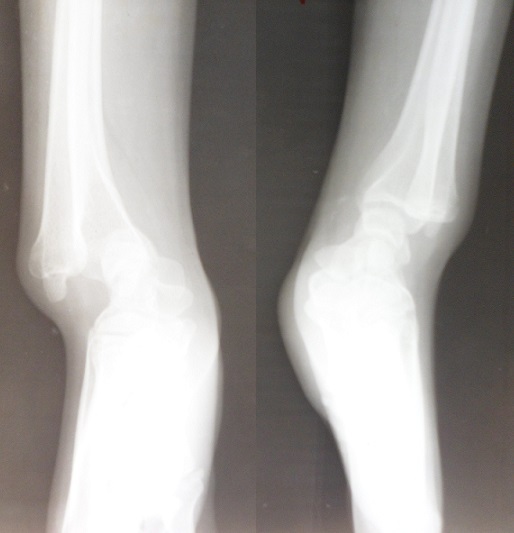
Radiographie comparative du poignet vue de profil

**Figure 5:**
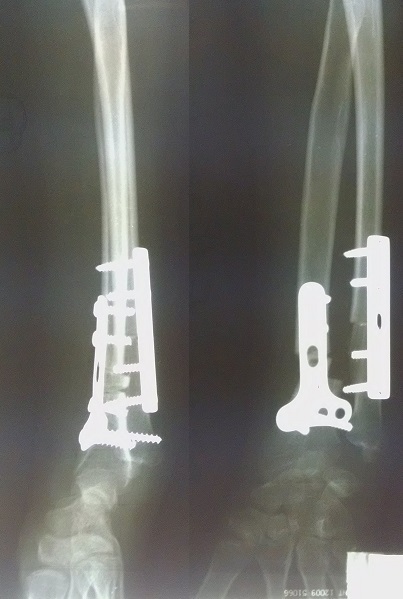
Radiographie de contrôle post opératoire immédiat

**Figure 6:**
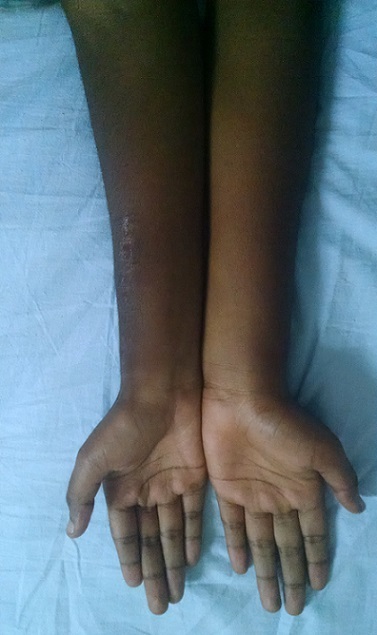
Radiographie de contrôle à la consolidation à 06 mois post opératoire

**Figure 7:**
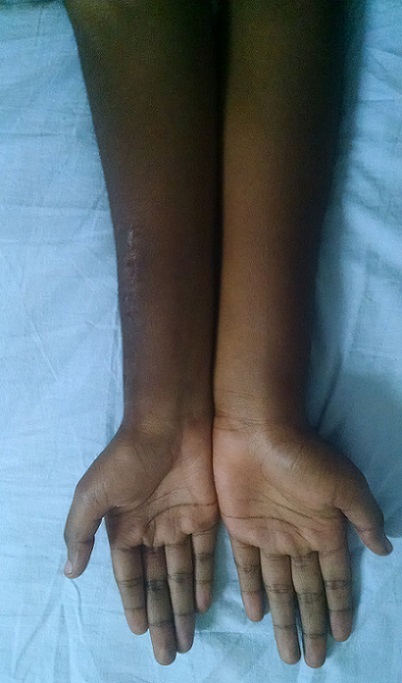
Aspect clinique en post opératoire avec un recul de 1 an 06 mois

Une patiente âgée de 21 ans de latéralité droite, sans antécédents particuliers, a consulté pour une déformation inesthétique douloureuse bilatérale des poignets, plus accentuée à droite évoluant depuis cinq ans. Cette douleur survient lors des activités quotidiennes de la vie courante et aussi lors des activités sportives. A L‘examen clinique, on a noté une saillie postérieurede la tête ulnairebilatérale (Figure 1), associée du côté droit à un déjettement palmaire du carpe et une courbure du radius (Figure 2). Les amplitudes articulaires des poignets étaient limitées à droit, concernant l'abduction, la supination et l'extension. La force de préhension était diminuée à droite. La radiographie comparative de l'avant-bras et du poignet incidence de face et profil objectivait des lésions caractéristiques de la déformation de Madelung (Figure 3 et Figure 4). Un traitement chirurgical a été indiqué et réalisé, une ostéotomie correctrice associée à une ostéosynthèse (Figure 5). La patiente est satisfaite au niveau de la déformation corrigée cliniquement (Figure 6), mais il persiste des épisodes de douleur lié à l'implant (Figure 7).

## Discussion

Décrite en 1878, la déformation de Madelung est une dyschondroplasie qui touche l'extrémité inférieure du radius et provoque une subluxation palmaire du carpe [3]. Le point de départ de la déformation est un dysfonctionnement du cartilage de conjugaison radial qui entraîne l'agénésie de la portion interne de l’épiphyse expliquant le radius curvus [4]. La déformation de Madelung apparait progressivement lors de la croissance et touche fréquemment la jeune adolescente, âgée de 8 à 13 ans, ayant une atteinte bilatérale rarement symétrique [2] comme c'est le cas dans notre observation. On distingue les formes, traumatiques, dysplasiques, idiopathiques et génétiques [5]. La déformation de Madelung, associée à une mésomélie et une petite taille définit le syndrome de Léri-Weill ou dyschondrostéose lié à une mutation du gène SHOX (*hort stature homeobox*, OMIM 127300) [6].

D'autres syndromes ont été également rapportés en association (syndrome de Turner, exostose multiple, maladied'Ollier ou achondroplasie) [7]. Enfin, les formes post-infectieuses ou post-traumatiques sont considérées par certains comme une déformation symptomatique (pseudo-Madelung) à distinguer de la forme idiopathique [8]. La première consultation est motivée par une douleur du poignet essentiellement à l'effort, mais également par une gêne esthétique due à la saillie de la tête ulnaire (au niveau des tissus mous) [3–8] très visible sur la Figure 1. Cette déformation devient évidente ou s'aggrave en fin de croissance [4]. Cliniquement, la déformation de Madelung se présente comme une subluxation dorsale du poignet par rapport au carpe qui semble déjeté vers l'avant [4]. L'aspect du profil est caractéristique visualisant la saillie postérieure de la tête ulnaire. Au préjudice esthétique et aux douleurs, vient s'ajouter parfois une diminution de la force musculaire [3]. À l'examen, la mobilité du poignet apparaît limitée en extension, la tête ulnaire faisant buter en arrière, tandis que la flexion est au contraire augmentée. L'inclinaison radiale est diminuée par la courbure du radius qui limite la distance styloïde radiale-trapèze [4]. La pronation peut aussi être bloquée par la tête ulnaire mais de manière moins constante alors que la supination reste complète [4]. Le diagnostic de la déformation de Madelung repose sur la présentation clinique et radiologique [8]. Les examens radiologiques doivent comprendre au minimum des clichés de face et de profil des poignets et des avant-bras [9] comme c'est le cas dans notre observation. Des critères radiologiques permettent de confirmer le diagnostic de la déformation de Madelung [7]. Les traitements conservateurs n'ont pas fait preuvede leur efficacité [7–10]. Le traitement est essentiellement chirurgical et de nombreuses techniques ont été décrites, nécessitant deprendre en compte l’âge du patient, la sévérité des symptômes etles objectifs souhaités (amélioration esthétique, traitement de ladouleur, amélioration des amplitudes en relation avec les activitésquotidiennes et les attentes du patient) car toutes les interventionsn'apportent pas les mêmes résultats [8].

## Conclusion

La déformation de Madelung est une affection rare, elle est diagnostiquée sur des critères cliniques et radiologiques. Le traitement chirurgical a prouvé son efficacité, mais ne doit pas être systématique.
