# Erratum: déformation de Madelung à propos d’un cas et revue de la littérature

**DOI:** 10.11604/pamj.2016.24.160.10103

**Published:** 2016-06-24

**Authors:** The Pan African Medical Journal

**Affiliations:** 1 The Pan African Medical Journal, Uganda, Kampala

**Keywords:** Erratum, maladie de Madelung, ulna, radius, Erratum, malnutrition, children, infections, infant mortality

## Abstract

Cet erratum corrige l’article original: “ Déformation de Madelung à propos d’un cas et revue de la littérature ”. The Pan African Medical Journal. 2016;23:137. doi:10.11604/pamj.2016.23.137.9002.

## Abstract (en)

This erratum corrects: “Infectious profile and mortality of children aged 0-5 years admitted with severe acute malnutrition: a retrospective cohort study conducted in the Therapeutic Nutritional Center in Bukavu, Republic of Congo”. The Pan African Medical Journal. 2016;23:137. doi:10.11604/pamj.2016.23.137.9002.

## Erratum

Dans la version originale de ce manuscrit [1] le tableau 1 été omis. Cela a été corrigé à la fois dans la version PDF et HTML dudit manuscrit.
